# Treatment Outcomes of Simple and Complex Central Serous Chorioretinopathy

**DOI:** 10.3390/jcm14051458

**Published:** 2025-02-21

**Authors:** Hiroyuki Kamao, Katsutoshi Goto, Tatsuhiro Ouchi, Yuki Shirakawa, Ryutaro Hiraki, Kenichi Mizukawa, Atsushi Miki

**Affiliations:** 1 Department of Ophthalmology, Kawasaki Medical School, 577 Matsushima, Kurashiki 701-0114, Japan; k_goto@med.kawasaki-m.ac.jp (K.G.); ouchi@mw.kawasaki-m.ac.jp (T.O.); wolfyurian0612kwsk@gmail.com (Y.S.); hiraki@med.kawasaki-m.ac.jp (R.H.); amiki@med.kawasaki-m.ac.jp (A.M.); 2 Shirai Eye Hospital, 1339 Takasecho Kamitakase, Mitoyo 767-0001, Japan; mizu-p@shirai-hosp.or.jp

**Keywords:** central serous chorioretinopathy, photocoagulation, photodynamic therapy, macular neovascularization

## Abstract

**Objectives**: To assess the association between clinical outcomes and the multimodal imaging-based classification of central serous chorioretinopathy (CSC). **Methods**: This retrospective study included 207 eyes from 155 treatment-naïve patients with CSC. The eyes were categorized into two groups, including the simple CSC group (*n* = 164) and the complex CSC group (*n* = 43), based on the presence of retinal pigment epithelial atrophy spanning two or more disc areas. All patients were initially observed without treatment for 3–6 months. For cases with persistent subretinal fluid after this observation period, treatment modalities, including continued observation, photocoagulation (PC), or photodynamic therapy (PDT), were selected. **Results**: Patients in the complex CSC group were more likely to be older (*p* = 0.01) and male (*p* = 0.01) than those in the simple group and to exhibit a higher prevalence of bilateral involvement (*p* < 0.001) and previous CSC episodes (*p* < 0.001) than those exhibited by patients in the simple group. Eyes with complex CSC exhibited a comparable incidence of spontaneous resolution within 6 months and a higher incidence of recurrence after spontaneous resolution within 6 months than eyes with simple CSC. In both the simple and complex CSC groups, the PDT subgroup exhibited a lower recurrence rate than that of the PC subgroup (simple CSC: *p* < 0.001, complex CSC: *p* = 0.03). **Conclusions**: Although CSC is typically a self-limiting disease often managed conservatively, patients with complex CSC, characterized by bilateral involvement or a history of previous episodes, are at a higher risk of subretinal fluid recurrence and may benefit from early interventions without a period of observation, such as PDT.

## 1. Introduction

Central serous chorioretinopathy (CSC), which predominantly affects middle-aged men, is characterized by subretinal fluid (SRF) associated with choroidal abnormalities, including filling delay of choroidal arteries and choriocapillaris, dilation of choroidal veins, and choroidal vascular hyperpermeability [1]. The increasing recognition of CSC in recent years was prompted by Fung et al.’s report on macular neovascularization (MNV) secondary to CSC [2]. Since this report, it has been recognized that neovascular age-related macular degeneration in Asian populations is often associated with MNV secondary to the characteristic choroidal abnormalities observed in CSC, leading to the establishment of the pachychoroid spectrum disease concept [3]. CSC is recognized as a representative phenotype within the pachychoroid spectrum diseases; however, the pathogenesis underlying MNV development from CSC remains unclear, necessitating a deeper understanding of CSC.

CSC is generally classified into two subtypes, including acute CSC and chronic CSC, based on the duration of SRF [4]. Acute CSC typically resolves spontaneously within 3 to 6 months, with a favorable visual prognosis. In contrast, chronic CSC is characterized by persistent SRF for more than 6 months, which can lead to permanent damage to the retinal pigment epithelium (RPE) and outer retinal atrophy, resulting in vision loss [5]. Consequently, recurrent and persistent SRF requires treatment, including laser photocoagulation (PC) [6], photodynamic therapy (PDT) [7], subthreshold micropulse laser [8], and selective retina therapy [9]. The choice of treatment methods are considered by factors such as the location of the fluorescent leakage, the fluorescent leakage pattern (focal or diffuse), and feasibility of off-label use. Accurate subtype classification is crucial for elucidating pathophysiology, predicting prognosis, and determining optimal treatment strategies. However, distinguishing between acute CSC and chronic CSC can be challenging because the duration of SRF often relies on subjective symptoms. A previous study demonstrated that patients with chronic CSC describe a relatively recent disease onset, despite having multimodal imaging findings indicating prolonged SRF [10]. To address this, the Central Serous Chorioretinopathy International Group recently proposed a new multimodal imaging-based classification system, classifying CSC into two subtypes: simple CSC and complex CSC. This classification is based on the presence or absence of RPE alterations larger than two-disc areas [11]. Previous report showed this classification achieves in good inter-physician agreement [12]. Additionally, previous reports demonstrated that patients with complex CSC are more likely to be older and male and exhibit bilateral involvement, poorer visual acuity, and thinner retinal thickness compared to those with simple CSC [13,14]. Nevertheless, few studies have explored clinical outcomes based on this classification.

In this study, we aimed to investigate the association between clinical outcomes and the multimodal imaging-based classification of CSC. Specifically, we analyzed patients with CSC who were initially observed without treatment for 3 to 6 months and subsequently treated with observation, photocoagulation (PC), or photodynamic therapy (PDT) in cases of unresolved SRF, focusing primarily on the resolution and recurrence of SRF.

## 2. Materials and Methods

### 2.1. Study Design

We retrospectively studied consecutive treatment-naïve patients with CSC at the Kawasaki Medical School between April 2009 and April 2024. Data on hypertension, diabetes, and cigarette smoking were collected from hospital or patient records. The collected data included age, sex, date of onset, previous CSC episodes, history of corticosteroid use, and ophthalmic treatment. Data on previous CSC episodes were obtained from patient recall and not from imaging findings. Patients were classified as never-smokers and ever-smokers, as described in a previous report [15].

All participants underwent a complete ophthalmologic examination, including refraction measurements, measurements of best-corrected visual acuity (BCVA), indirect ophthalmoscopy, slit-lamp biomicroscopy with a noncontact lens, color fundus photography and fundus autofluorescence (FAF) (TRC-50DX; Topcon corporation, Tokyo, Japan), spectral domain optical coherence tomography (OCT) (RS-3000 Advance 2 OCT; Nidek Corporation, Gamagori, Japan), swept-source OCT (DRI OCT-1 Atlantis; Topcon Corporation, Tokyo, Japan), fluorescein angiography (FA), and indocyanine green angiography (ICGA) (HRA-2; Heidelberg Engineering GmbH, Dossenheim, Germany). The spherical equivalent (SE) was determined by adding half of the cylindrical power to the primary spherical power. Visual acuity data were obtained as decimal visual acuity (BCVA) values and converted to the logarithm of the minimum angle of resolution (logMAR) units for analysis. Central retinal thickness (CRT) and subfoveal choroidal thickness (SFCT) were measured using swept-source OCT, as previously described [16]. We measured the BCVA, CRT, and SFCT in eyes with CSC at the initial visit, the resolution of retinal exudate, and the final visit to determine the treatment outcomes. Pachydrusen was defined as yellow-white deposits on color fundus photographs, corresponding to sub-RPE accumulation above the pachyvessels on OCT images. Pachydrusen showed a hyperfluorescent area on mid- to late-phase ICGA. Among drusen showing hyperfluorescence on the ICGA, those smaller than 125 µm in size were classified as punctate hyperfluorescence spots (PHSs) [17]. PHSs are typically scattered as single or clustered hyperfluorescent spots. Eyes with resolved CSC (CSC without SRF) and/or flat, irregular PED were excluded. A previous study reported that one-third of patients with CSC and flat irregular PED had macular neovascularization (MNV) [18]; therefore, they were excluded from the present study. Other exclusion criteria included a history of intraocular surgery, PC, PDT, or anti-VEGF therapy; previous or current systemic corticosteroid therapy; history of systemic conditions associated with CSC; pregnancy; and other retinal and choroidal diseases.

### 2.2. Diagnosis and Classification of CSC

**Figure 1:**
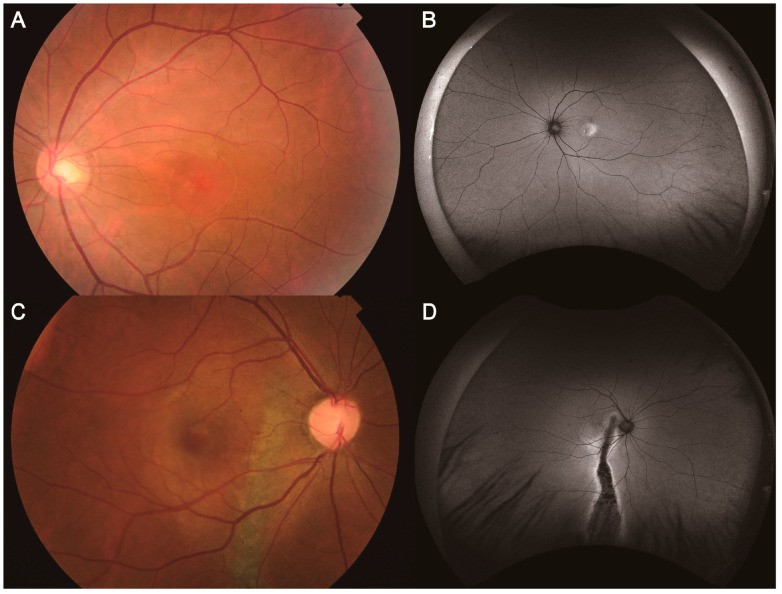
A representative case of the left eye of a 34-year-old man with simple CSC, with a SE of 1.5 diopter (**A**,**B**). (**A**) Color fundus photography revealed serous retinal detachment in the macula. (**B**) FAF demonstrated hyperfluorescence corresponding to subretinal fluid. Based on the FAF findings, the hyperfluorescent area was ≤1 disc diameter, and no hypofluorescent areas were observed; therefore, we classified this case as simple CSC. A representative case of the left eye of a 43-year-old man with complex CSC, with a SE of 0.5 diopter (**C**,**D**). (**C**) Color fundus photography revealed serous retinal detachment in the macula and RPE alteration. (**D**) FAF demonstrated hypoautofluorescence with surrounding hyperautofluorescence as a descending tract. Based on the FAF findings, the hypofluorescent area was >5 disc diameter; therefore, we classified this case as complex CSC. CSC: central serous chorioretinopathy, SE: spherical equivalent, FAF: fundus autofluorescence, RPE: retinal pigment epithelium.

The diagnosis and classification of CSC were conducted according to the criteria described in a previous report [10]. The major diagnostic criteria for CSC included (1) the presence or evidence of previous SRF in the posterior pole on OCT images and (2) one or more RPE changes detected on FAF and OCT images. The minor diagnostic criteria for CSC consisted of (1) choroidal vascular hyperpermeability on ICGA images, (2) one or more focal leakages on FA images, or (3) SFCT greater than 400 µm on OCT images, adjusted for age and axial length. CSC was diagnosed when both the major and at least one of the minor diagnostic criteria were met. CSCs were classified into simple and complex CSC based on the extent of RPE atrophy detected on FAF and OCT images. Simple CSC was defined as eyes with a total RPE atrophy area equal to or less than two-disc areas, whereas complex CSC was defined as eyes with a total RPE atrophy area greater than two-disc areas (Figure 1).

### 2.3. Treatment Method

All patients were initially observed without treatment for 3–6 months after their first visit to our hospital. For cases with unresolved SRF after this observation, treatment modalities, including continued observation, PC, or PDT, were selected based on the patient’s circumstances and FA and ICGA findings. PC was performed using a 577 nm wavelength, 200 μm spot size, 0.2 s duration, and 80–100 mW power, producing a light grey burn at the fluorescence leakage point. PDT was administered using the half-dose verteporfin method (3 mg/m^2^) with standard parameters: fluency of 50 J/cm^2^, wavelength of 689 nm, and treatment duration of 83 s. Verteporfin was infused for over 10 min, and PDT was performed 15 min after the start of infusion. The PDT laser spot size was determined based on the diameter of the affected area identified by FA and ICGA, with an added safety margin of 500 µm. This affected area included all leakage areas observed on FA as well as some choroidal vascular hyperpermeability areas observed on ICGA.

### 2.4. Statistical Analysis

Statistical analyses were conducted using the JMP Pro 17.0.0 software (SAS Institute, Cary, NC, USA). The Kruskal–Wallis test was used to compare age, SE, BCVA, CRT, SFCT, and follow-up duration between the two study groups. Pearson’s chi-square test was used to analyze differences in the female-to-male ratios, bilateral involvement, hypertension, diabetes, ever-smokers, previous CSC episodes, PHS, pachydrusen, intraretinal fluid (IRF), resolution, and recurrence of SRF. Statistical significance was set at *p* < 0.05.

## 3. Results

### 3.1. Patient Characteristics

A total of 207 eyes (simple CSC: 164; complex CSC: 43) from 155 treatment-naïve patients with CSC were included in our study. The baseline characteristics of these patients are summarized in Table 1. Patients in the complex CSC group were older (*p* = 0.01), less likely to be female (*p* = 0.01), and exhibited a higher proportion of bilateral involvement, previous CSC episodes (*p* < 0.001), and IRF (*p* = 0.02) than were those in the simple CSC group. The two study groups were comparable in terms of the prevalence of hypertension, diabetes, smoking habits, PHS, and pachydrusen. Eyes in the complex CSC group were also associated with hyperopia (*p* < 0.001), worse BCVA (*p* < 0.001), thinner CRT (*p* = 0.005), and thicker SFCT (*p* = 0.06) than were those in the simple CSC at the initial visit.

### 3.2. Treatment Outcomes Between the Simple CSC and Complex CSC Groups

**Figure 2:**
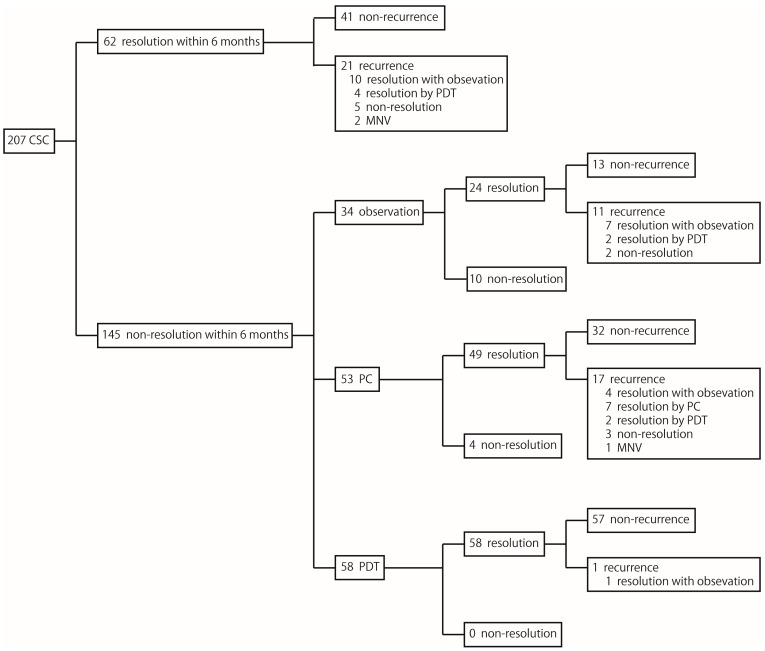
Treatment profiles of patients with CSC.

Treatment outcomes were compared between the simple CSC and complex CSC groups in terms of resolution and non-resolution of SRF within 6 months (Table 2). The non-resolution of SRF within the 6-month group included patients treated with continued observation, PC, and PDT (Figure 2). No significant difference was observed between the simple and complex CSC groups in spontaneous resolution of SRF within 6 months (31.1% vs. 25.6%; *p* = 0.48). However, among those who achieved spontaneous resolution, the recurrence rate was significantly higher in the complex CSC group than in the simple CSC group (63.6% vs. 27.5%; *p* = 0.03). No significant difference was observed in the distribution of treatment modalities between the two study groups among patients whose SRF did not resolve spontaneously within 6 months (*p* = 0.58). Additionally, no significant difference was observed in the resolution of SRF between the two study groups in the continued observation (*p* = 0.58), PC (*p* = 0.14), and PDT (*p* = 1.00) subgroups. Similarly, no significant difference was found in the recurrence rate after SRF resolution between the two study groups in the continued observation (*p* = 0.51), PC (*p* = 0.86), and PDT (*p* = 0.44) subgroups. Baseline and final visit assessments indicated that complex CSC was associated with worse BCVA and thinner CRT than was simple CSC across all subgroups, including those with SRF resolution within 6 months. However, no differences were observed in the mean changes in BCVA, CRT, or SFCT between the two study groups across all subgroups.

### 3.3. Treatment Outcomes Between the PC and PDT Subgroups

We compared treatment outcomes between the PC and PDT subgroups (Table 3). A significantly higher proportion of patients in the PDT subgroup achieved SRF resolution than those in the PC subgroup (100.0% vs. 92.5%; *p* = 0.01). Among the patients who achieved SRF resolution, the PDT subgroup achieved a significantly lower recurrence rate than that of the PC subgroup (1.7% vs. 34.7%; *p* < 0.001).

**Figure 3:**
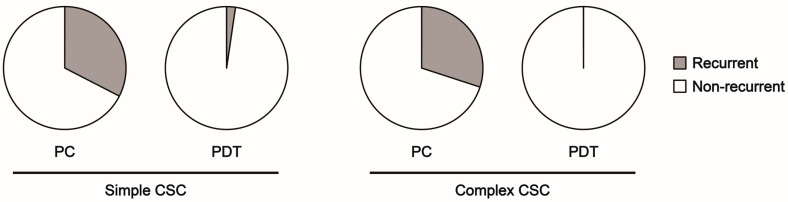
Recurrence rate of PC and PDT subgroups in the simple and complex CSC groups. The recurrence rate in the simple CSC group was 32.6% (14/43) in the PC subgroup and 2.3% (1/43) in the PDT subgroup (*p* < 0.001). The recurrence rate in the complex CSC group was 30.0% (3/10) in the PC subgroup and 0.0% (0/15) in the PDT subgroup (*p* = 0.03). CSC: central serous chorioretinopathy, PC: laser photocoagulation, PDT: photodynamic therapy.

**Figure 4:**
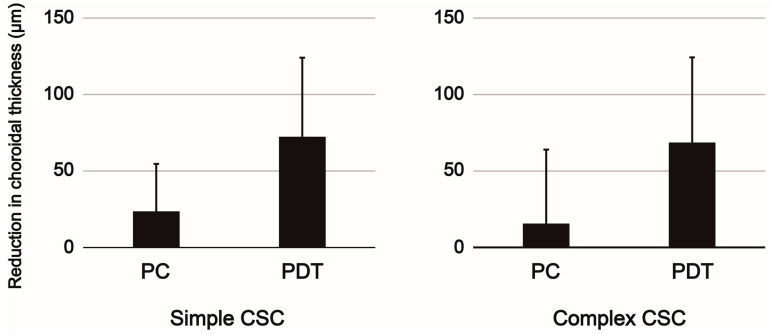
Reduction in choroidal thickness at the resolution of SRF in the PC and PDT subgroups in the simple and complex CSC groups. The reduction in choroidal thickness in the simple CSC group was 23.4 ± 31.3 µm in the PC subgroup and 72.1 ± 52.0 µm in the PDT subgroup (*p* < 0.001). The reduction in choroidal thickness in the complex CSC group was 15.3 ± 48.7 µm in the PC subgroup and 68.4 ± 55.9 µm in the PDT subgroup (*p* = 0.04). SRF: subretinal fluid, CSC: central serous chorioretinopathy, PC: laser photocoagulation, PDT: photodynamic therapy.

No differences were observed between the two study groups in terms of the mean BCVA at baseline or the mean change in BCVA at the time of SRF resolution. However, significant differences were identified between the two study groups in the mean CRT and SFCT at baseline and in the mean change in CRT and SFCT at the time of SRF resolution. Multivariable logistic regression analysis was then conducted, incorporating the recurrence rate after resolution of SRF, CRT, and SFCT at baseline and the mean change in CRT and SFCT at the time of SRF resolution. It revealed that the PDT subgroup had a significantly lower recurrence rate (*p* < 0.001) and greater reduction in SFCT (*p* < 0.001) than did the PC subgroup. The finding that the PDT subgroup exhibited a lower recurrence rate and greater reduction in SFCT than the PC subgroup was consistent in both the simple and complex CSC groups (Figure 3 and Figure 4).

### 3.4. Incidence of MNV in Eyes with CSC

**Figure 5:**
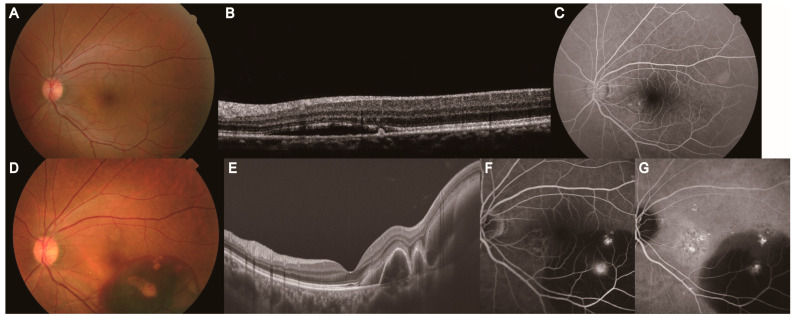
Development of MNV in a 42-year-old man with simple CSC within the 6-month resolution group. Images (**A**–**C**) represent images 71 months before the development of MNV. Images (**D**–**G**) represent images at MNV onset. (**A**) Color fundus photograph revealed serous retinal detachment in the nasal fovea. (**B**) B-scan with a spectral domain OCT image of the macula showed SRF with PED. (**C**) Early-phase FA image showed the area of hyperfluorescence (leakage point) in the nasal fovea. (**D**) Color fundus photograph revealed retinal hemorrhage with yellowish lesions in the temporal–inferior fovea. (**E**) B-scan with a swept-source OCT image of the macula showed steep PED with subretinal hyperreflective material (subretinal hemorrhage). (**F**) Late-phase FA image showed areas of hyperfluorescence corresponding to yellowish lesions and hypofluorescence corresponding to retinal hemorrhage in the temporal–inferior fovea. Late-phase FA image revealed the hyperfluorescent area in the nasal fovea corresponding to the previous CSC leakage point. (**G**) Late-phase ICGA image showed areas of hyperfluorescence indicating polypoidal lesions in the temporal-inferior fovea and hyperfluorescence corresponding to choroidal vascular hyperpermeability in the nasal fovea. CSC: central serous chorioretinopathy, MNV: macular neovascularization, OCT: optical coherence tomography, ICGA: indocyanine green angiography, FA: fluorescein angiography, PED: pigment epithelial detachment.

**Figure 6:**
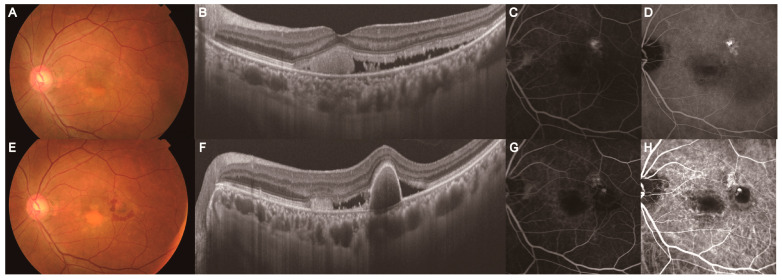
Development of MNV in a 53-year-old man with simple CSC within the 6-month resolution group. Images (**A**–**D**) represent images 86 months before the development of MNV. Images (**E**–**H**) represent images at MNV onset. (**A**) Color fundus photograph revealed yellowish lesions with serous retinal detachment in the fovea. (**B**) B-scan with a swept-source OCT image of the macula showed subretinal fluid with subretinal hyperreflective material (exudation). (**C**) Late-phase FA image showed the area of hyperfluorescence as a CSC leakage point in the temporal–superior fovea. (**D**) Late-phase ICGA image showed areas of hyperfluorescence as choroidal vascular hyperpermeability in the temporal-superior fovea. (**E**) Color fundus photograph revealed an orange-reddish lesion with retinal hemorrhage in the temporal fovea and yellowish lesions in the macula. (**F**) B-scan with a swept-source OCT image of the macula showed steep PED with subretinal fluid and hyperreflective material (exudation). (**G**) Late-phase FA image showed areas of hyper- and hypofluorescence in the temporal fovea. (**H**) Late-phase ICGA image revealed a hyperfluorescent area indicating a polypoidal lesion in the temporal fovea, located adjacent to a hyperfluorescent area corresponding to the previous CSC leakage point. CSC: central serous chorioretinopathy, MNV: macular neovascularization, OCT: optical coherence tomography, ICGA: indocyanine green angiography, FA: fluorescein angiography, PED: pigment epithelial detachment.

**Figure 7:**
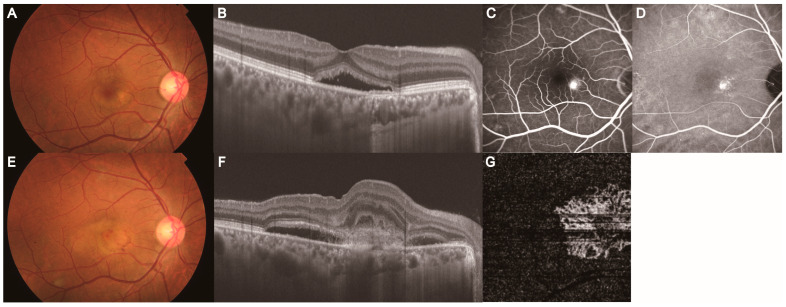
Development of MNV in a 37-year-old man with simple CSC in the PC group. Images (**A**–**D**) represent images 97 months before MNV development. Images (**E**–**G**) represent images at MNV onset. (**A**) Color fundus photograph revealed serous retinal detachment in the fovea. (**B**) B-scan with a swept-source OCT image of the macula showed subretinal fluid. (**C**) Mid-phase FA image showed the area of hyperfluorescence as a CSC leakage point in the nasal fovea. (**D**) Mid-phase ICGA image showed areas of hyperfluorescence as choroidal vascular hyperpermeability in the nasal fovea. (**E**) Color fundus photograph revealed a yellow-white lesion with retinal hemorrhage in the nasal fovea. (**F**) B-scan with a swept-source OCT image of the macula showed subretinal fluid and hyperreflective material with the disruption of RPE/Bruch’s membrane. (**G**) OCTA image showed a visualized vascular structure corresponding to subretinal hyperreflective material. CSC: central serous chorioretinopathy, MNV: macular neovascularization, OCT: optical coherence tomography, ICGA: indocyanine green angiography, FA: fluorescein angiography, PED: pigment epithelial detachment.

We analyzed the 207 eyes with CSC to evaluate the incidence of MNV. During a mean follow-up period of 26.8 ± 35.4 months (6.25–179.5 months), MNV developed in three eyes in the simple CSC group, while no cases were observed in the complex CSC group. No significant difference was observed in the incidence of MNV between the simple CSC and complex CSC groups (*p* = 0.24). The treatment modalities in the three eyes that developed MNV were as follows: two eyes were in the SRF resolution within 6 months group (Figure 5 and Figure 6) and one eye was in the PC subgroup (Figure 7). Two eyes in the 6-month resolution group developed type 1 MNV with polypoidal lesions at 71 and 86 months after the initial visit, separately. The one eye in the PC subgroup developed type 2 MNV at 97 months after the initial visit. In the 6-month resolution group, one MNV lesion developed in an area adjacent to the fluorescence leakage point, and the other lesion developed in an area distant from the fluorescence leakage point. In the PC subgroup, the MNV lesion developed within the fluorescence leakage point.

## 4. Discussion

Our study revealed that eyes with complex CSC exhibited worse visual acuity, a comparable proportion of spontaneous resolution of SRF within 6 months, and a higher proportion of recurrence after spontaneous resolution of SRF within 6 months compared to eyes with simple CSC. Additionally, complex CSC was associated with a higher prevalence of bilateral involvement and previous CSC episodes than that of simple CSC. To the best of our knowledge, no prior studies have investigated the recurrence rate after spontaneous resolution in simple and complex CSC. These findings suggest that eyes with complex CSC are prone to frequent recurrence after spontaneous resolution, which may lead to outer retinal atrophy and subsequent visual impairment. Although CSC is generally a self-limiting disease and is often managed conservatively, patients with complex CSC characteristics, such as bilateral involvement or a history of previous CSC episodes, are at high risk of SRF recurrence and may benefit from early treatment.

In this study, patients with complex CSC were characterized by older age, male sex, previous CSC episodes, bilateral involvement, hyperopia, worse visual acuity, thinner retina, thicker choroid, and the presence of IRF. These findings are consistent with the findings of previous studies investigating CSC characteristics using this multimodal imaging-based classification [13,14,19]. Additionally, Imanaga et al. reported that patients with complex CSC had thicker sclera, and Yoneyama et al. found that complex CSC was associated with distinct genetic characteristics [13,14]. Furthermore, Arora et al. examined 229 patients with CSC and reported that patients with complex CSC were more likely to exhibit poor visual acuity, recurrent CSC episodes, persistent SRF, and outer retinal atrophy [19]. Although these findings align with our results, their complex CSC group showed a higher prevalence of persistent SRF, whereas our study demonstrated a similar proportion of spontaneous resolution within 6 months between simple and complex CSC. This discrepancy may be attributed to differences in study cohorts, as the study by Arora et al. included patients with CSC who received previous or current corticosteroid therapy. Based on the duration of SRF, CSCs are commonly classified into two subtypes: acute CSC and chronic CSC. However, a previous study has highlighted a high degree of discordance in this classification among experienced retina specialists [20]. In contrast, the classification of CSC into simple CSC and complex CSC demonstrated moderate consensus among retina physicians at different institutions [12] and high agreement among retina physicians at the same institution [21]. Furthermore, the clinical characteristics of patients with CSC using this classification, as observed in previous studies and our study, are generally consistent, supporting the clinical utility of this multimodal imaging-based classification. It should be noted that previous CSC episodes in this study were determined based solely on patient recall, without considering the presence or absence of signs of previous SRF. This approach differs from the diagnostic methods used in previous studies, which classified CSC based on multimodal imaging findings. Patient recall is prone to underestimation, as previous studies have shown that patients with chronic CSC often report a relatively recent onset of symptoms despite multimodal imaging findings indicating prolonged SRF [10]. However, unlike multimodal imaging, patient recall has the advantage of simplicity and does not require specialized equipment. Despite these differences in diagnostic methods, our findings are consistent with those of previous studies showing that the proportion of patients with previous CSC episodes was higher in the complex CSC group, emphasizing the significance of patient recall in the clinical assessment of CSC.

No reports have directly compared treatment outcomes between simple CSC and complex CSC. This study found that the treatment effects of PC and PDT, including changes in visual acuity and retinal thickness, the proportion of SRF resolution, and the recurrence rate after SRF resolution, were comparable between simple CSC and complex CSC. In contrast, the PDT subgroup demonstrated higher treatment efficacy, as evidenced by the higher proportion of resolution and the lower proportion of recurrence after resolution than those associated with the PC subgroup. Numerous studies have evaluated treatment outcomes in patients with CSC. However, no consensus has been reached regarding the standard therapy for CSC, largely because CSC often resolves spontaneously and has a relatively favorable visual prognosis, even if left untreated in many cases. To date, two randomized controlled trials have evaluated the therapeutic efficacy of PDT in patients with CSC [22,23]. The PLACE trial compared the half-dose PDT group with a high-intensity subthreshold micropulse laser treatment (HSML) group in 179 patients with chronic CSC and demonstrated the superiority of the half-dose PDT group in achieving SRF resolution [22]. The SPECTRA trial assessed a half-dose PDT group versus an oral eplerenone group in 107 patients with chronic CSC, highlighting the superiority of the half-dose PDT group in the resolution of SRF [23]. Furthermore, regarding recurrence after SRF resolution, the PLACE trial reported that the half-dose PDT group had a significantly lower recurrence rate than that of the HSML group (7% vs. 47%) one year after the resolution of SRF. Lai et al. compared the half-dose PDT group to the placebo group in 192 patients with CSC, reporting a significantly lower recurrence rate in the half-dose PDT group than that in the observation without treatment group (20% vs. 54%) after more than 3 years of follow-up following SRF resolution [24]. Several studies comparing the treatment outcomes of PDT and PC have demonstrated the superiority of PDT. The full-dose PDT group exhibited a more rapid resolution of SRF (1.2 months vs. 1.8 months) and a lower recurrence rate (10% vs. 30%) compared to the PC group [25]. Additionally, another study reported that the rate of dry macula at 3 months post-treatment was significantly higher with half-dose PDT than with PC [26]. CSC has recently garnered attention as a representative pachychoroid disease, with choroidal vascular hyperpermeability and congestion playing critical roles in its pathogenesis [27]. PDT reduces choroidal thickness to relatively normal levels in patients with unilateral CSC and reduces choroidal vascular hyperpermeability [28]. In contrast to PDT, PC in patients with CSC did not result in significant changes in SFCT [29]. In the present study, the PDT subgroup exhibited a significantly greater reduction in choroidal thickness after treatment compared to the PC subgroup. This finding suggests that PDT improved choroidal vascular hyperpermeability and congestion, which are the underlying causes of CSC, thereby contributing to the lower recurrence rate observed in the PDT subgroup. Regarding long-term treatment outcomes of PDT, Park et al. reported an improvement in mean BCVA by 0.15 logMAR [30], and Haga et al. reported an improvement of 0.13 logMAR [31]. Although few studies have evaluated treatment outcomes of CSC classified into simple and complex CSC, our study demonstrated a BCVA improvement of 0.07 logMAR in simple CSC and 0.06 logMAR in complex CSC, supporting the efficacy of PDT in alignment with previous reports. Although the treatment methods differed, our findings are consistent with those of previous studies, reinforcing the notion that PDT remains the most effective modality for treating patients with CSC.

MNV is a significant cause of visual impairment in CSC. Before the establishment of a new disease entity called pachychoroid neovasculopathy (PNV) [32], the development of MNV in patients with CSC was primarily attributed to type 2 MNV, often resulting from previous laser treatment [33]. However, with the recognition of PNV, it has become widely accepted that type 1 MNV can develop in patients with CSC due to prolonged SRF or persistent choroidal circulatory insufficiency. In this study, MNV developed in two cases following spontaneous resolution and in one case after resolution with PC. The MNV that developed after spontaneous resolution was identified as type 1 MNV and was observed at a location distinct from the fluorescence leakage site associated with CSC. Previously, we evaluated the clinical characteristics of PHS in patients with unilateral MNV and no drusen in the fellow eye. We found that in cases where MNV developed in the fellow eye with PHS, the MNV emerged at a location distinct from the PHS area. Similarly, Teo et al. evaluated patients with unilateral MNV and found that MNV did not co-localize with pachydrusen when MNV developed in the fellow eye [34]. In contrast, the MNV that developed after resolution by PC occurred at the fluorescence leakage site where PC was performed. These results indicate that the development of MNV after spontaneous resolution and resolution by PC is different, not only in the MNV subtype but also in the location of the MNV. Furthermore, all three cases in this study involved patients with simple CSC, demonstrating that the development of MNV in patients with CSC does not necessarily require progression through complex CSC.

One limitation of this study includes its retrospective design, which resulted in a lack of standardization of treatment strategies and follow-up protocols for patients who did not achieve spontaneous resolution within 6 months. Treatment methods tended to be selected depending on the pattern of fluorescent leakage. Patients with focal leakage were more likely to undergo PC, whereas those with diffuse leakage were more often treated with PDT. Although this study confirmed no significant difference in the frequency of treatment methods (observation, PC, and PDT) between patients with simple CSC and complex CSC, the possibility of a selection bias between PC and PDT could not be excluded. Conducting a prospective study with standardized treatment protocols and follow-up procedures would help minimize biases and provide more reliable results. Additionally, examining long-term outcomes beyond 6 months would provide a better understanding of the sustainability of SRF resolution and recurrence rates. The concept of pachychoroid diseases, represented by CSC, has been established as a cause of MNV. However, the mechanism underlying the development of MNV is not fully elucidated. In this study, two cases of MNV were observed in patients with simple CSC, while no cases were found in those with complex CSC. Comparing MNV associated with simple CSC and complex CSC in the future may help clarify this process of PNV development. Thus, large-scale and long-term follow-up studies are needed.

## 5. Conclusions

Although CSC is typically a self-limiting disease often managed conservatively, patients with bilateral involvement or a history of previous episodes, characteristic of complex CSC, are at a higher risk of subretinal fluid recurrence. Therefore, these patients may benefit from early interventions without a period of observation, such as PDT.

## Figures and Tables

**Table 1:** Baseline characteristics of patients with CSC.

	Simple CSC (*n* = 164)	Complex CSC (*n* = 43)	*p*
Age (years), mean (SD)	52.2 (12.9)	58.1 (13.7)	0.01
Sex (female), no. (%)	30 (18.3)	2 (4.7)	0.01
Bilateral, no. (%)	26 (15.9)	27 (62.8)	<0.001
Hypertension, no. (%)	41 (25.0)	16 (37.2)	0.12
Diabetes, no. (%)	15 (9.1)	8 (18.6)	0.10
Smoking habits (ever-smokers), no. (%)	99 (60.4)	34 (79.1)	0.052
Previous CSC episodes (recurrent), no. (%)	51 (31.1)	32 (74.4)	<0.001
PHS, no. (%)	123 (75.0)	37 (86.0)	0.20
Pachydrusen, no. (%)	21 (12.8)	10 (23.3)	0.10
IRF, no. (%)	4 (2.4)	5 (11.6)	0.02
Spherical equivalent (diopters), mean (SD)	−0.23 (1.99)	1.13 (1.45)	<0.001
Baseline, mean (SD)			
BCVA (logMAR)	0.01 (0.20)	0.22 (0.28)	<0.001
CRT (µm)	368.4 (127.3)	306.6 (102.2)	0.005
SFCT (µm)	373.5 (123.6)	420.7 (137.4)	0.06

CSC: central serous chorioretinopathy, SD: standard deviation, PHS: punctate hyperfluorescence spots, IRF: intraretinal fluid, BCVA: best-corrected visual acuity, CRT: central retinal thickness, SFCT: subfoveal choroidal thickness.

**Table 2:** Treatment outcomes between the simple CSC and complex CSC groups.

	Resolution Within 6 M			Non-Resolution Within 6 M								
				Observation			PC			PDT		
	Simple CSC	Complex CSC	*p*	Simple CSC	Complex CSC	*p*	Simple CSC	Complex CSC	*p*	Simple CSC	Complex CSC	*p*
Eyes, no. (%)	51 (31.1)	11 (25.6)	0.48	27 (23.9)	7 (21.9)		43 (38.1)	10 (31.3)		43 (38.1)	15 (46.9)	0.58
Resolved, no. (%)	-	-	-	18 (66.7)	6 (85.7)	0.58	41 (95.4)	8 (80.0)	0.14	43 (100.0)	15 (100.0)	1.00
Recurrent, no. (%)	14 (27.5)	7 (63.6)	0.03	8 (29.6)	3 (42.9)	0.51	14 (34.2)	3 (37.5)	0.86	1 (2.3)	0 (0.0)	0.44
Baseline, mean (SD)												
BCVA (logMAR)	−0.02 (0.18)	0.21 (0.30)	0.004	0.00 (0.19)	0.26 (0.44)	0.08	0.02 (0.20)	0.26 (0.30)	0.004	0.07 (0.22)	0.18 (0.20)	0.04
CRT (µm)	365.2 (129.0)	309.2 (152.0)	0.16	330.0 (111.1)	292.5 (92.5)	0.61	412.2 (134.3)	312.9 (97.9)	0.02	352.6 (119.1)	306.1 (68.9)	0.19
SFCT (µm)	386.4 (124.7)	468.3 (112.5)	0.10	362.1 (126.0)	338.0 (92.3)	0.76	416.3 (130.5)	473.7 (158.1)	0.36	358.7 (117.9)	430.9 (125.9)	0.09
Final visit, mean (SD)												
BCVA (logMAR)	−0.06 (0.20)	0.17 (0.34)	0.004	0.00 (0.21)	0.23 (0.22)	0.02	−0.04 (0.16)	0.26 (0.54)	0.049	0.00 (0.20)	0.12 (0.23)	0.04
CRT (µm)	223.3 (41.6)	208.6 (103.7)	0.10	240.6 (46.5)	170.5 (38.6)	0.004	217.0 (53.2)	176.5 (44.8)	0.04	196.1 (27.8)	172.5 (31.3)	0.008
SFCT (µm)	365.4 (112.5)	400.0 (163.7)	0.50	334.4 (114.0)	274.8 (121.2)	0.51	386.6 (128.6)	403.0 (130.9)	0.81	283.6 (125.9)	357.9 (150.5)	0.10
Change, mean (SD)												
BCVA (logMAR)	0.03 (0.15)	0.04 (0.15)	0.92	0.00 (0.13)	0.03 (0.31)	0.66	0.06 (0.17)	0.00 (0.32)	0.80	0.07 (0.13)	0.06 (0.10)	0.80
CRT (µm)	141.9 (131.6)	100.5 (166.4)	0.38	89.4 (112.8)	122.0 (99.8)	0.51	195.2 (145.7)	136.4 (78.5)	0.20	156.5 (119.3)	133.7 (65.9)	0.67
SFCT (µm)	21.0 (34.2)	68.3 (132.8)	0.87	33.7 (31.4)	63.2 (29.0)	0.07	29.8 (36.4)	70.7 (131.6)	0.90	75.2 (53.5)	73.0 (61.7)	0.90
Follow-up month, mean (SD)	20.2 (27.0)	15.9 (17.5)	0.98	51.6 (43.6)	66.1 (65.1)	0.50	27.6 (35.1)	49.3 (67.3)	0.45	15.3 (12.6)	21.1 (16.6)	0.003

CSC: central serous chorioretinopathy, PC: laser photocoagulation, PDT: photodynamic therapy, SD: standard deviation, BCVA: best-corrected visual acuity, CRT: central retinal thickness, SFCT: subfoveal choroidal thickness.

**Table 3:** Treatment outcomes between the PC and PDT subgroups.

	PC (*n* = 53)	PDT (*n* = 58)	Univariate *p*	Multivariate *p*
Time of SRF resolution, month, mean (SD)	3.1 (2.8)	1.7 (0.5)	0.65	
Recurrent, no. (%)	17 (34.7)	1 (1.7)	<0.001	<0.001
Baseline, mean (SD)				
BCVA (logMAR)	0.06 (0.24)	0.10 (0.22)	0.23	
CRT (µm)	397.7 (136.0)	340.6 (109.8)	0.01	0.69
SFCT (µm)	431.3 (131.2)	373.8 (121.2)	0.045	0.07
Change at the resolution of SRF, mean (SD)				
BCVA (logMAR)	0.06 (0.16)	0.04 (0.14)	0.84	
CRT (µm)	201.0 (133.8)	150.5 (108.1)	0.02	0.83
SFCT (µm)	22.1 (34.0)	71.3 (52.3)	<0.001	<0.001

PC: laser photocoagulation, PDT: photodynamic therapy, SD: standard deviation, BCVA: best-corrected visual acuity, CRT: central retinal thickness, SFCT: subfoveal choroidal thickness.

## Data Availability

The data used to support the findings of this study are restricted by the Kawasaki Medical School Ethics Committee to protect patient privacy. Data are available from Hiroyuki Kamao (hironeri@med.kawasaki-m.ac.jp) for researchers who meet the criteria for access to confidential data.
